# Management of Severe Penile and Scrotal Degloving Injury Following Dog Bite in an Infant: A Case Report

**DOI:** 10.1002/ccr3.72952

**Published:** 2026-06-15

**Authors:** Denis Mucunguzi, Marvin Mwesigwa Mutakooha, Mawejje Saul, Kwesiga Divine, Donald Dominick Lema, Alex Mremi

**Affiliations:** ^1^ Department of Surgery Mbarara University of Science and Technology Mbarara Uganda; ^2^ Division of Urology Mbarara University of Science and Technology Mbarara Uganda; ^3^ School of Medicine, KCMC University Moshi Tanzania; ^4^ Department of Pathology Kilimanjaro Christian Medical Centre Moshi Tanzania; ^5^ Department of Research and Training Kilimanjaro Clinical Research Institute Moshi Tanzania

**Keywords:** dog bite, pediatric genital trauma, phalloplasty, rabies prophylaxis, surgical reconstruction, urethral injury

## Abstract

Dog bites are a common cause of pediatric trauma, although genital involvement is rare and presents significant reconstructive challenges. We report a 7‐month‐old male infant with severe penile and scrotal degloving injury and distal urethral involvement following a domestic dog bite. Following stabilization, the patient underwent irrigation, debridement, urethral catheterization, and staged reconstruction including delayed scrotal closure and penile reconstruction with a full‐thickness skin graft. Ceftriaxone antibiotics and rabies post‐exposure prophylaxis were administered. Recovery was uneventful, and at one‐month follow‐up, the child demonstrated satisfactory wound healing and preserved urinary function without fistula or stricture. This case highlights the importance of early wound management, infection prophylaxis, individualized reconstruction, and long‐term follow‐up to monitor delayed functional and cosmetic outcomes.

## Key Clinical Message

Severe pediatric genital dog‐bite injuries, though rare, require prompt irrigation, debridement, antimicrobial and rabies prophylaxis, and individualized reconstruction. Early surgical intervention with urethral stenting may preserve urinary function and cosmetic outcomes, while long‐term follow‐up is essential to detect late complications affecting urethral patency, penile growth, function, and appearance.

## 1 Introduction

Dog bites represent a major global public health concern and disproportionately affect children [1]. Although craniofacial injuries account for the majority of dog‐bite trauma in children under 5 years of age, genital involvement is uncommon but potentially devastating [2]. Published literature, based on a structured search of indexed databases (PubMed/Medline) and a narrative synthesis of available case reports and small case series, identifies fewer than 25 reported pediatric cases of genital dog‐bite injuries to date. These reports represent an aggregate of individual case reports rather than a single formal systematic review, with clinical presentations ranging from superficial lacerations to complete penile amputation or bilateral testicular loss [3].

Genital dog bites pose unique surgical and reconstructive challenges due to the delicate anatomy, risk of infection, and potential long‐term functional and psychosocial consequences [3]. Management requires prompt trauma assessment, meticulous wound care, prophylaxis against tetanus and rabies where indicated, and timely reconstructive intervention tailored to the extent of tissue loss. In infants, preservation of urinary function and normal genital development is of paramount importance.

We report a rare case of severe penile and scrotal degloving injury with distal urethral involvement in a 7‐month‐old infant following a domestic dog bite. Given the scarcity of published pediatric cases describing both the extent of genital soft tissue loss and urethral involvement, there remains a limited understanding of optimal reconstructive approaches in this setting. The aim of this report is therefore to describe the surgical management strategy employed and to present the short‐term functional and anatomical outcomes following reconstruction.

## 2 Case History/Examination

A 7‐month‐old male infant was brought to the emergency department approximately 24 h after sustaining genital injuries from a bite by a domestic household dog while playing at home. According to the mother, the child had frequent close interaction with the pet. There was no reported loss of consciousness or additional trauma. The dog's rabies vaccination status could not be confirmed at presentation. The injury was therefore classified as a WHO Category III exposure due to transdermal bites with severe tissue injury and mucosal/urogenital involvement, warranting immediate post‐exposure prophylaxis. In addition, the wound was consistent with a contaminated traumatic bite injury (CDC/IDSA Class III/dirty wound), given the devitalized tissue and high risk of polymicrobial infection.

## 3 Methods (Differential Diagnosis, Investigations and Treatment)

On examination, the infant was irritable and in obvious discomfort but hemodynamically stable. There were no associated injuries. Local examination revealed extensive soft tissue loss involving the ventral aspect of the penis, partial loss of the glans, degloving of the distal penile shaft, and a hanging scrotal skin flap. The distal urethral meatus was poorly visualized, raising concern for urethral injury. Both testes were palpable within the scrotum.

Baseline hematological investigations demonstrated leukocytosis (white blood cell count 20.54 × 10^3^/μL) with marked lymphocytosis (absolute lymphocyte count 16.56 × 10^3^/μL; 80.6%), while neutrophil counts remained within the normal range. Hemoglobin, red blood cell indices, platelet count, and other differential parameters were largely within age‐appropriate reference ranges, with no clinically significant abnormalities identified. Following stabilization, the patient was taken to the operating theater for examination under general anesthesia.

**FIGURE 1:**
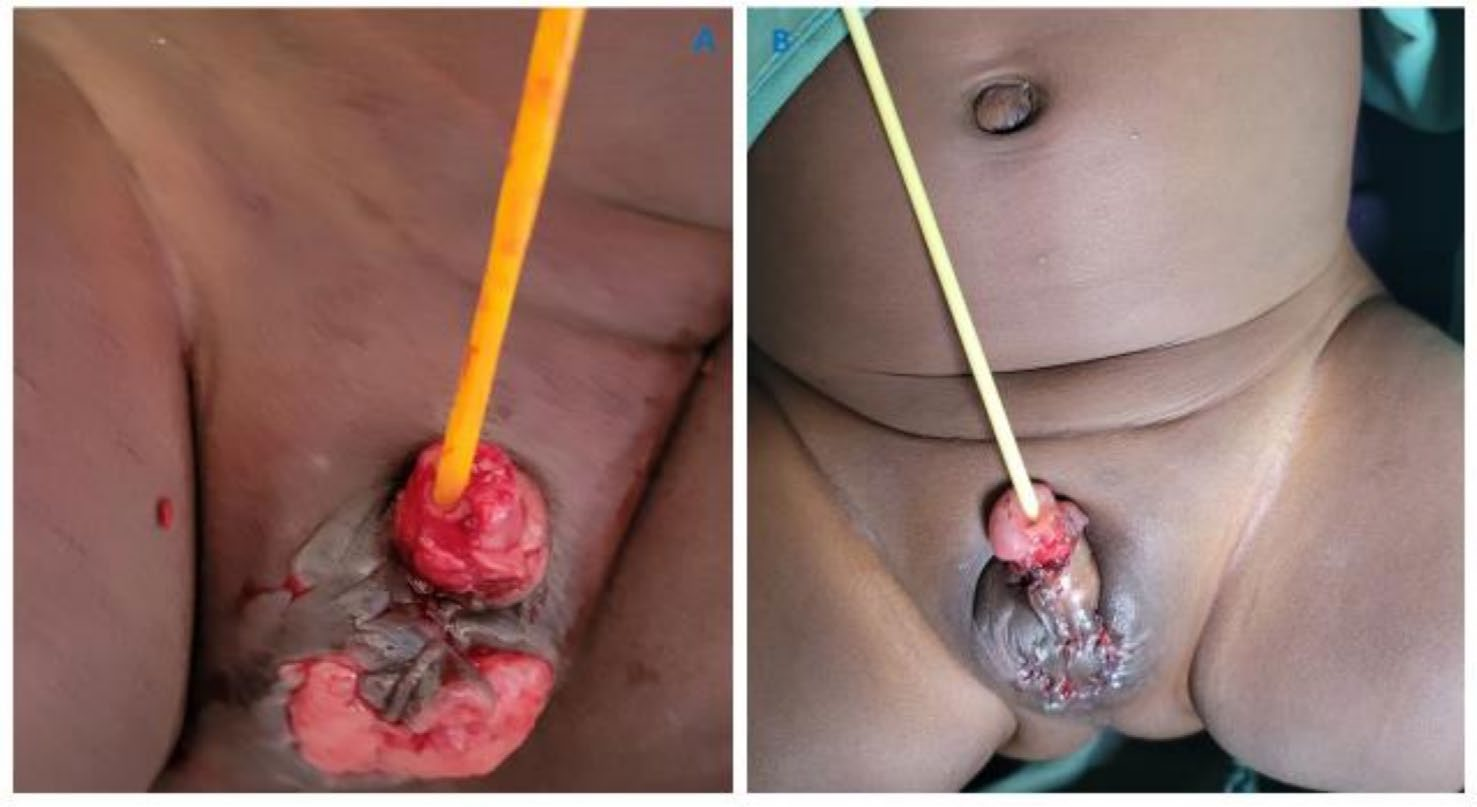
Genital injuries at initial presentation in the emergency room (A) and on the first postoperative day following genital reconstruction with primary closure and full‐thickness skin grafting (B).

Intraoperative findings confirmed significant loss of ventral penile skin and glanular tissue with distal urethral injury. Thorough wound irrigation with copious normal saline and meticulous debridement of devitalized tissue were performed. An 8F Foley catheter was gently inserted to stent the urethra and ensure urinary diversion (Figure 1A). Broad‐spectrum intravenous antibiotics at a dose of 50 mg/kg once daily for 1 week were initiated with ceftriaxone as the primary agent to provide coverage against common dog oral flora, including *Pasteurella multocida* and *Capnocytophaga canimorsus* , as well as other mixed aerobic and anaerobic organisms typically associated with bite wound infections. This choice was made in view of the high‐risk nature of the contaminated genital injury and the potential for rapid polymicrobial soft tissue infection. Rabies post‐exposure vaccination was administered on days 0, 3, 7, and 14 according to national protocol.

Human rabies immunoglobulin (HRIG) infiltration was indicated in this case, given the WHO Category III exposure characterized by a transdermal bite with severe tissue injury. However, HRIG was not administered due to unavailability at our facility at the time of presentation. This omission carries a recognized risk of suboptimal passive immunization in Category III exposures; nevertheless, the patient received immediate wound care and rabies vaccination as per protocol to mitigate the risk of viral transmission.

Given the extent of tissue loss and contamination risk, delayed primary closure of the scrotal wound was performed. Penile shaft reconstruction was performed using a full‐thickness skin graft harvested from the inguinal region, selected for its color and texture match to genital skin. The graft was tailored to cover the defect (approximately 2 × 1 cm) and was secured in place using interrupted absorbable sutures to ensure precise edge approximation and minimize graft shear. A tie‐over dressing was applied to provide uniform pressure and enhance graft adherence. Postoperatively, the graft was immobilized with a non‐adherent dressing and close monitoring was undertaken for signs of hematoma, infection, or graft take, with regular wound inspections during the early healing phase. Hemostasis was ensured, and sterile dressings were applied.

## 4 Conclusions and Results (Outcome and Follow‐Up)

**FIGURE 2:**
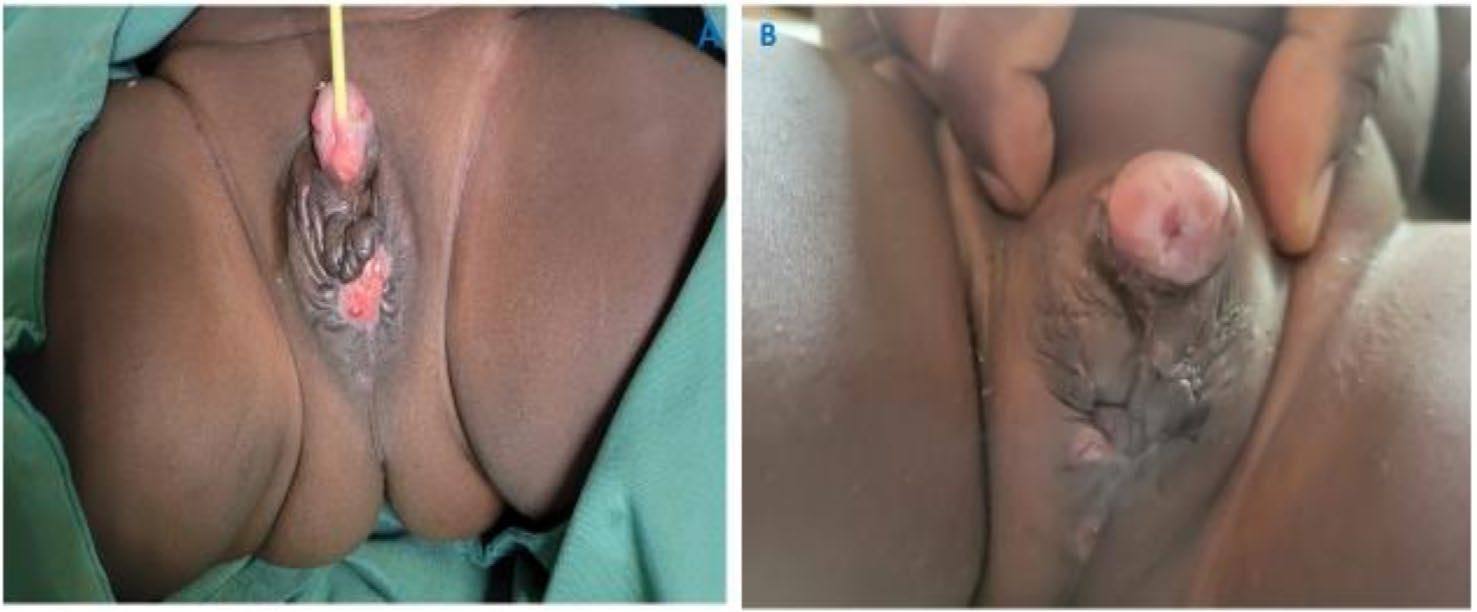
Patient presentation after 1 week (A) and 1 month (B) of daily wound care.

**FIGURE 3:**
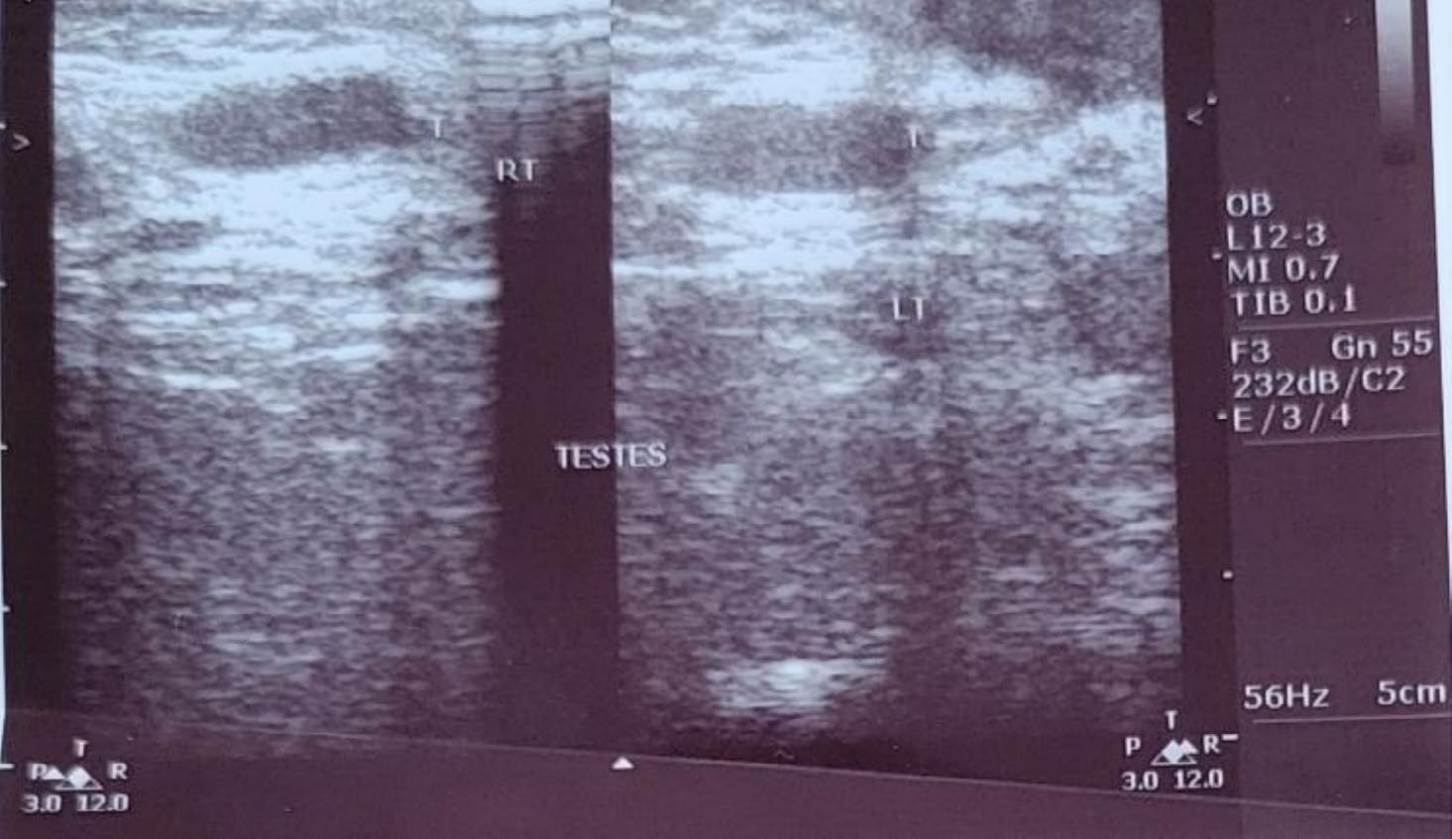
Follow‐up scrotal ultrasound showing both testes correctly positioned within the scrotum.

The postoperative course was uneventful (Figure 1B). Daily wound care was conducted, and the urethral catheter was maintained for 10 days (Figure 2A). On removal of the catheter, the infant voided with a single, forward urinary stream. At one‐month follow‐up, early wound healing was satisfactory with an acceptable initial cosmetic appearance and no immediate postoperative complications (Figure 2B). Both testes remained in the scrotum on clinical and ultrasonographic evaluation. No evidence of urethrocutaneous fistula, meatal stenosis, or wound infection was observed (Figure 3). However, given the short duration of follow‐up, definitive assessment of long‐term outcomes—particularly urethral patency, risk of stricture formation, penile growth, and functional results—cannot yet be established. No validated outcome scoring systems or imaging studies (e.g., urethrography) were performed at this early stage, and further longitudinal follow‐up is planned to objectively evaluate anatomical and functional integrity over time.

## 5 Discussion

Although dog bites are common in pediatric populations, genital involvement remains rare [1]. Most reported pediatric cases involve males, likely reflecting anatomical exposure [3]. The spectrum of injury ranges from minor lacerations to complete genital destruction. In severe cases, loss of glans tissue, urethral injury, or bilateral orchiectomy has been described [4].

Initial management follows established standard trauma principles based on Advanced Trauma and Life Support (ATLS) prioritizing hemodynamic stability and comprehensive injury assessment [5]. In genital dog bites, early and copious irrigation is critical to reduce bacterial load and minimize infection risk. Dog oral flora includes mixed aerobic and anaerobic organisms; therefore, broad‐spectrum antibiotic coverage is recommended [6]. Prophylaxis against tetanus and rabies must be considered according to local epidemiology and exposure risk.

Primary closure of bite wounds remains controversial due to infection concerns [6]. However, evidence suggests that with adequate debridement and early presentation, selected wounds may undergo primary or delayed primary closure without increased infection risk [6]. In our patient, delayed scrotal closure and staged reconstruction were chosen due to the extent of contamination and tissue loss.

Reconstruction of penile degloving injuries requires restoration of both function and cosmesis [7]. Options include split‐thickness skin grafts, full‐thickness grafts, or local flaps depending on available tissue and defect size [8]. In infants, tissue availability and long‐term growth potential must be considered. Urethral stenting may be considered in selected cases with urethral involvement to support alignment, facilitate urinary drainage, and potentially reduce the risk of stricture formation; however, its indication is case‐dependent and should be individualized based on the extent of injury and intraoperative findings [9].

Potential long‐term complications include meatal stenosis, urethral stricture, fistula formation, penile curvature, and adverse psychosocial outcomes [10]. Although early results in our case were favorable, prolonged follow‐up through childhood and puberty will be necessary to assess genital growth and functional development.

This case highlights the clinical and logistical challenges of managing severe Category III rabies exposure and complex genital trauma in a low‐resource setting, particularly the unavailability of Human Rabies Immunoglobulin (HRIG), which is recommended by WHO guidelines for immediate passive immunization in all Category III exposures alongside vaccine administration. In comparison with WHO standards and previously reported pediatric cases, where HRIG administration and structured reconstructive protocols are more consistently available, our case demonstrates a gap in adherence driven by resource constraints rather than clinical indication. In this patient, efforts were made to explore alternative access through referral pathways to nearby designated centers; however, these attempts were unsuccessful due to logistical constraints and stock unavailability. The patient therefore received wound management and rabies vaccination according to national guidelines, but without immunoglobulin administration. Strengthening supply chains and preventive public health strategies remains crucial in endemic regions.

Notably, this case is distinguished by the very young age of the patient (7 months), the combined severity of penile and scrotal degloving with distal urethral involvement, and the use of a full‐thickness inguinal skin graft for reconstruction in an emergency setting. These factors, together with limited access to HRIG and advanced reconstructive adjuncts, underscore important implications for strengthening rabies prophylaxis availability and surgical capacity within similar resource‐limited health systems in line with WHO rabies prevention and control guidelines.

## 6 Conclusion

Severe genital dog‐bite injuries in infants are rare but potentially devastating. Prompt stabilization, meticulous wound irrigation and debridement, appropriate antimicrobial and rabies prophylaxis, and individualized reconstructive planning are essential to optimize outcomes. Early surgical intervention can preserve urinary function and acceptable cosmesis. Long‐term follow‐up is necessary to monitor for urethral complications and ensure normal genital development.

## Author Contributions

**Denis Mucunguzi:** conceptualization, project administration, writing – original draft, writing – review and editing. **Marvin Mwesigwa Mutakooha:** resources, supervision. **Mawejje Saul:** data curation, investigation, resources. **Kwesiga Divine:** data curation, investigation, resources. **Donald Dominick Lema:** resources, writing – review and editing. **Alex Mremi:** data curation, methodology, supervision, writing – review and editing.

## Funding

The authors have nothing to report.

## Ethics Statement

Ethical approval for the case report is exempt at our institution. Consent from the patient is considered sufficient for writing a case report.

## Consent

Written informed consent was obtained from the patient for publication of this case report and accompanying images. A copy of the written consent is available for review by the Editor‐in‐Chief of this journal on request.

## Conflicts of Interest

The authors declare no conflicts of interest.

## Data Availability

The data that support the findings of this study are available on request from the corresponding author. The data are not publicly available due to privacy or ethical restrictions.
